# Tuning the Immune Cell Response through Surface Nanotopography Engineering

**DOI:** 10.1002/smsc.202400227

**Published:** 2024-07-21

**Authors:** Raïssa Rathar, David Sanchez‐Fuentes, Hugo Lachuer, Valentin Meire, Aude Boulay, Rudy Desgarceaux, Fabien P. Blanchet, Adrian Carretero‐Genevrier, Laura Picas

**Affiliations:** ^1^ Institut de Recherche en Infectiologie de Montpellier (IRIM) Université de Montpellier CNRS UMR 9004 Montpellier 34000 France; ^2^ Institut d’Électronique et des Systèmes (IES) Université de Montpellier CNRS UMR 5214 Montpellier 34000 France; ^3^ CNRS Université de Paris Institut Jacques Monod 75013 Paris France

**Keywords:** actin organization, dendritic cells, membrane remodeling, nanoimprint lithography, nanostructured surfaces, sol–gel chemistry

## Abstract

Dendritic cells (DCs) are central regulators of the immune response by detecting inflammatory signals, aberrant cells, or pathogens. DC‐mediated immune surveillance requires morphology changes to adapt to the physical and biochemical cues of the external environment. These changes are assisted by a dynamic actin cytoskeleton–membrane interface connected to surface receptors that will trigger signaling cascades. In recent years, the development of synthetic immune environments has allowed to investigate the impact of the external environment in the immune cell response. In this direction, the bioengineering of functional topographical features should make it possible to establish how membrane morphology modulates specific cellular functions in DCs. Herein, the engineering of one‐dimensional nanostructured SiO2 surfaces by soft‐nanoimprint lithography to manipulate the membrane morphology of ex vivo human DCs is reported. Super‐resolution microscopy and live‐cell imaging studies show that vertical pillar topographies promote the patterning and stabilization of adhesive actin‐enriched structures in DCs. Furthermore, vertical topographies stimulate the spatial organization of innate immune receptors and regulate the Syk‐ and ERK‐mediated signaling pathways across the cell membrane. In conclusion, engineered SiO_2_ surface topographies can modulate the cellular response of ex vivo human immune cells by imposing local plasma membrane nano‐deformations.

## Graphical abstract

Dendritic cells (DCs) are central regulators of the host's immune response against pathogens and cancerous cells. Soft‐nanoimprint lithography is used to engineer one‐dimensional nanostructured SiO_2_ surfaces to tune the spatial organization of the actin cytoskeleton, immune receptors, and signaling molecules in ex vivo human DCs and, hence, selectively modulate the immune cell response through the environment topography.

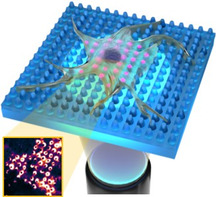

## 1 Introduction

The innate immune system, the first line of defense of the body, is made to react against external agents, including viruses, bacteria, and cancerous cells. This central function is fulfilled by cells of the myeloid lineage, including macrophages, neutrophils, dendritic cells (DCs), or natural killer cells, through a process known as immune surveillance.^[^ ^1^ ^]^ During this process, cells migrate to inflammation sites, kill pathogens, and resolve the infection locally by releasing cytokines and recruiting cells from the innate and adaptative immune response. Then, antigen‐presenting cells (APCs) can also migrate to the lymph nodes and initiate the adaptive immune response.^[^ ^2^ ^]^ Immune surveillance will, however, force myeloid cells to encounter different environmental cues, including chemokines and rigidity gradients, topographical features, pressure gradients, or a given molecular composition of the extracellular matrix.^[^ ^3^ ^]^

In latest years, bioengineering of synthetic immune environments has been instrumental in examining the interaction of immune cells with their environment and addressing how external cues influence their behavior.^[^ ^3^, ^4^, ^5^ ^]^ In this line, one of the main challenges is the development of innovative surfaces allowing, for instance, to selectively modulate specific cell‐fate decisions, trigger specific pro‐ or anti‐inflammatory responses, or potentiate cell survival and proliferation. To date, most of the current developments in this direction have focused on the micro‐ and nanofabrication of protein patterns on rigid and soft surfaces^[^ ^5^, ^6^, ^7^ ^]^ and the engineering of 2D microtextured surfaces and microfabricated geometries made of soft polymeric materials, such as polydimethylsiloxane (PDMS), polyacrylamide (PAA) Dimethyl sulfoxide or poly(d,l‐lactic‐co‐glycolic acid (PLGA), allowing to investigate on the traction forces exerted by cells.^[^ ^3^, ^8^, ^9^ ^]^ Conversely, the engineering of rigid topographical features, which instead probe the local effect of the membrane morphology, in the context of the immune cell response is still an emerging field.^[^ ^3^ ^]^ Yet, micro‐ and nanoscale surface topographies have shown to be a powerful approach to investigating topography‐mediated cellular responses on epithelial cells and fibroblasts, from the biochemical transduction of curvature‐sensing proteins^[^ ^10^, ^11^, ^12^, ^13^ ^]^ to nuclear transduction.^[^ ^14^ ^]^ The fabrication of topography‐based synthetic environments can be achieved through top‐down approaches, such as focused ion beam microscopy or electron beam lithography,^[^ ^11^, ^15^, ^16^ ^]^ although bottom‐up approaches, such as soft‐gel nanoimprint lithography (soft‐NIL) would be the strategy of choice to engineer large‐area micro‐ and nano‐topographies.^[^ ^12^ ^]^

Why is it relevant to investigate the local effect of topographical features and the response of myeloid cells to plasma membrane deformations? In myeloid cells, central immune processes, such as migration, pathogen capture, or immune synapse formation, are powered by the plasma membrane deformation through its association with the actin cytoskeleton.^[^ ^17^ ^]^ Furthermore, the association of the plasma membrane with an underlying cortex of filamentous actin (F‐actin) also regulates the spatial organization and lateral diffusion of immune receptors,^[^ ^18^, ^19^ ^]^ which might then support pathogen recognition and capture. Of note, immune functions that originate at the plasma membrane/actin cortex interface rely on the function of integral membrane receptors and, in addition, to the local binding of cytosolic proteins in response to signaling events, ultimately leading to changes in gene expression.^[^ ^20^, ^21^ ^]^ Hence, engineering synthetic immune environments to investigate the plasma membrane response of human myeloid cells to local topographical features is central to understanding and eventually modulate their immune surveillance function.

In this study, we employed a bottom‐up large‐area micro‐ and nanofabrication methodology. We engineered 1D vertical pillar SiO_2_ nanostructured surface topographies by soft‐NIL, which allowed us to manipulate the local plasma membrane morphology. This manipulation served as a strategy to modulate the cellular response of ex vivo human myeloid cells selectively. Our focus was on human DCs, which are cells of the myeloid lineage functioning as phagocytes and APCs and endowed with potent migratory capacities. These cells play a central role in initiating the immune response by acting as messengers between innate and adaptive immunity. To observe the DCs response, we used nanostructured surfaces in combination with super‐resolution microscopy and live‐cell imaging. Our results show that local topographic cues prompt the formation and stabilization of patterned adhesive actin‐enriched structures at the plasma membrane of immature DCs, resembling hollow podosomes. We found that topography‐induced patterning of micron‐sized actin‐rich structures arises from a combination of repulsion, at short scales, and clustering at the whole cell level. This phenomenon involves the Syk‐mediated signaling and is convoyed by the patterning of defined immune receptors at the surface of DCs. Furthermore, we show that topographic cues modulate extracellular signal‐regulated kinases (ERK) energy Dispersive X‐ray phosphorylation and translocation into the nucleus. In conclusion, our study provides a detailed understanding of the mechanisms by which engineered SiO_2_ nanostructured topographies can be used to modulate the cellular response of human DCs through changes in the physical cues exerted by the external environment.

## 2 Results and Discussion

### 2.1 Engineered 1D Nanostructured Topographies Promote the Patterning of Actin‐Enriched Adhesive Structures in Human Dendritic Cells

**Figure 1:**
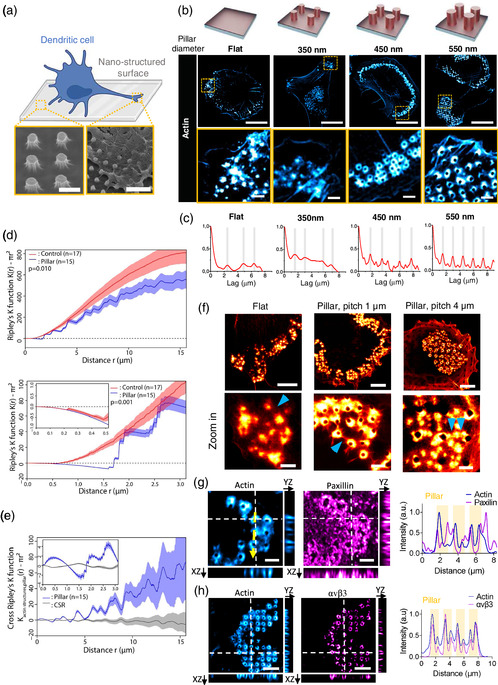
Spatial organization of adhesive actin‐rich structures on hDCs seeded on vertical nanostructured topographies. a) Immature hDCs cultured on SiO_2_ vertical pillar arrays engineered by soft‐NIL. Scanning electron microscopy images showing a detail of the surface topography (left) and the deformation of the plasma membrane of hDCs to adapt the shape of the surface topography (right). Scale bar: 1.5 and 3 μm, respectively. Schematics generated by BioRender. b) Airyscan images of the F‐actin staining of hDCs cultured on flat or 1D nanostructured surfaces with pillar arrays of 350, 450 and 550 nm diameter. Scale bar, 20 μm. c) 2D autocorrelation function (ACF) of the F‐actin signal from hDCs seeded on flat or pillar arrays of different diameter. d) Average Ripley's K function ± SEM at different length scales (*r*) of hDCs seeded on flat SiO_2_ surfaces (control, red curve) and 550 nm diameter pillar array surfaces (pillar, blue curve). Cells (*n*) analyzed from > 3 biological replicates, *n* = 17 and *n* = 15, respectively. Black dashed line represents the expected pattern in the case of CSR. Permutation test between the two experimental conditions, *P* < 0.01 and *P* < 0.001, at *r* < 15 μm and *r* < 3 μm, respectively. e) Average cross‐K function ± SEM at different length scales (*r*) of hDCs seeded on 550 nm diameter pillar array surfaces (blue curve). Cells (*n*) analyzed from > 3 independent experiments, *n* = 15. Black curve represents the averaged Ripley's K function of simulated CSR actin distribution independent on the pillar's localizations. Gray shade represents envelope containing 95% of simulations. f) STED images showing the organization of F‐actin of hDCs cultured on flat or nanostructured surfaces with pillar arrays of 550 nm diameter with a 2 or 4 μm pitch. Blue arrows point to actin cables connecting micron‐sized actin‐rich structures. Scale bar: 10 μm. g,h) Z‐projected Airyscan images (XY plane) of hDCs cells cultured pillar arrays of 550 nm diameter showing the organization of F‐actin (blue) and paxillin (g, magenta) or *α*v*β*3 (h, magenta). Orthogonal view along the white dashed line displaying the XZ and YZ planes. Cross‐section analysis along the yellow dashed‐line in the corresponding image. Scale bar, 2 and 5 μm, respectively.

The actin cytoskeleton is the primary determinant of the plasma membrane architecture of immune cells as it assists pathogen uptake, cell migration, immune synapse formation and cytokine secretion, among other central processes.^[^ ^17^ ^]^ Therefore, we engineered different surface topographies to investigate how cortical actin responds to the local curvature of the external environment. To this end, we used ex vivo human monocytes derived into dendritic cells (moDCs, as detailed in the experimental section), hereafter called hDCs. Immature hDCs were then seeded on flat (control) and SiO_2_ engineered surfaces displaying arrays of vertical pillars over a range of different diameter (i.e., 350, 450, and 550 nm) and an aspect ratio of ≈2:1 (height : diameter) that we generated by soft‐nanoimprint lithography (**Figure** **1**, Figure S1a and Table S1, Supporting Information), as previously described.^[^ ^12^, ^22^ ^]^ Seeding of cells on SiO_2_ vertical topographies forced the plasma membrane to curve and follow the morphology of the underlying surface (Figure 1a). After 4 h of seeding on fibronectin‐coated SiO_2_ surfaces, we analyzed the actin organization on fixed cells by using subdiffraction airyscan microscopy (Figure 1b). We observed the presence of micron‐sized actin‐enriched structures at the interface between adhering hDCs and SiO_2_ engineered surfaces, for all the conditions tested. We found that micron‐sized actin structures consistently accumulated at specific regions of the cell irrespectively of the underlying topography (Figure 1b).

To statistically measure the contribution of the surface topography on the spatial organization of actin structures, we performed a two‐dimensional (2‐D) autocorrelation function (ACF) image analysis (Figure 1c). We found that the organization of micron‐sized actin structures showed a smooth transition from a complete lack of correlation, for vertical pillars of 350 nm, to a complete correlation for vertical pillars of 550 nm of diameter, as we observed a periodic organization that correlated with the expected spacing of the pillar array, i.e., ≈1.5 μm (Figure 1c). Hence, we focused our further investigations on the 550 nm diameter 1D vertical pillar arrays (hereafter called, vertical pillar topographies).

We reasoned that micrometric actin‐enriched structures could be considered as a pattern of points and asked if their spatial organization could result from a random process (complete spatial randomness, CSR) or, instead, reflect the existence of an aggregation or repulsion process between the points (i.e., actin structures). To statistically quantify the nature of the spatial pattern of actin as a result of engineered surfaces, we generated two datasets corresponding to the coordinates of micron‐sized actin structures of 15 cells seeded on flat surfaces or surfaces displaying 550 nm diameter vertical pillars. Next, we computed the Ripley's K function, wich quantifies the average number of neighboring points within a distance *r* of a randomly chosen point, for the two experimental conditions (Figure 1d). Hence, positive values of the function reveal attraction/clustering, and negative values indicate dispersing/repulsion. The average curve obtained from the experiential events was significantly different from the Monte Carlo simulated curve in the case of CSR, irrespective of the type of surface (flat or pillars) in which hDCs were cultured (Figure S2, Supporting Information). This result indicates the existence of a spatial pattern organization of micron‐sized actin structures already under control conditions. Indeed, we observed a preferential clustering of micron‐sized actin structures in the scale of the whole cell (i.e., up to 15  μm to avoid edge effects). The evaluation of shorter scales, *r* ≤ 3 μm, revealed a negative deviation from the Ripley's K function, indicating the existence of repulsion in the spatial pattern of micron‐sized actin structures. On flat surfaces, we found a dispersing/repulsion phenomenon up to *r* ≈0.5 μm, followed by a shift in the curve tendency toward positive values indicative of clustering. Hence, pointing out the existence of a minimal distance between neighboring micron‐sized actin structures that form at the interface of hDCs with the substrate. This minimal distance is probably due to a steric‐like repulsion. Hence, if structures are micron‐sized, it is unlikely to observe two points’ closers than this distance. Nevertheless, the distance obtained in hDCs is in agreement with the neighbor distance found between actin cores in osteoclasts and macrophages.^[^ ^23^, ^24^ ^]^ Noteworthy, on hDCs seeded on vertical pillar topographies, we found that this dispersing/repulsion phenomenon was maintained until *r* ≈1.5  μm, which matched with the estimated spacing between the engineered vertical pillars (Figure S1, Supporting Information).

To determine if the spatial patterns observed at short distances in hDCs seeded on engineered topography surfaces results from the association of actin structures with 550 nm diameter vertical pillars or, instead, that actin structures are statistically independent on pillar localizations, we computed the multivariate Ripley's K function (Figure 1e). Briefly, the cross‐K function provides information on the type of interaction of two type of points (*x* and *y*), in our case the coordinates of actin structures and vertical pillars, at distance *r*. We observed a deviation of the cross‐K function >*πr* ^2^ around ≈0.5 μm, indicating enrichment of actin stuctures around pillars. Then cross‐K function decreases until 1.5 μm, which fits with the expected pillar spacing demonstrating that we do not observe additional actin structures between pillars. Finally, we observed a second local maximum at 2 μm because the cross‐K starts to probe a second pillar (i.e., 2 μm corresponds to the spacing of 1.5 μm plus the clustering distance of 0.5 μm). As a result, local maxima/minima repeat periodically reflecting the periodicity of the pillar array. Collectively these results indicate seeding hDCs on 550 nm diameter pillar arrays promote the association and spatial pattering of actin‐enriched structures through the combination of different effects at different length scales in a periodic manner: i) in the submicrometric range assisting the association of actin‐enriched structures; and ii) in the micrometric range promoting repulsion between adjacent rich‐actin structures due to the pillar spacing.

The results above indicate a direct effect of the vertical pillar size and spacing on actin‐enriched structure patterning. Thus, we asked how different pillar pitches might affect the spatial organization of these rich F‐actin structures in hDCs. To this end, we engineered SiO_2_ surfaces displaying 550 nm diameter 1D vertical pillars arrays with a pitch of ≈2 and 4 μm (Figure S1b and Table S1, Supporting Information) and we analyzed the actin organization of hDCs by super‐resolution STED microscopy (Figure 1f). We found that micron‐sized actin structures appeared grouped at specific regions, independently of the pillar pitch. Furthermore, we observed the presence of actin cables connecting actin structures in all the conditions tested. Interestingly, engineering of large ≈ 4 μm pitch pillar arrays prompted the appearance of two actin‐enriched structure organizations co‐existing in the same cell: one organized around the pillar and another within the pillar's spacing.

The formation of micron‐sized actin‐enriched structures at the adhesion interface of monocyte‐derived cells (such as macrophages, osteoclasts or immature DCs) with the substrate are named “podosomes” owing to their molecular organization.^[^ ^24^ ^]^ These types of structures fulfill central roles in adhesion, migration, mechanosensing, and phagocytosis.^[^ ^25^ ^]^ These micron‐sized rich actin structures are typically made of a core of branched F‐actin that is surrounded by a ring of *β*2 and *β*3 integrins, and adhesion molecules such as talin, paxillin, and vinculin.^[^ ^24^, ^26^ ^]^ Thus, we set out to determine the presence of adhesive molecules characteristic of podosomes in immature hDCs (Figure S3, Supporting Information). To this end, we performed immunofluorescence assays on hDCs seeded on SiO_2_ surfaces displaying 550 nm diameter vertical pillar arrays to visualize the localization of endogenous paxillin and integrin *β*3 (Figure 1g and h). We found that both adhesive proteins co‐localized with patterned actin structures and that their signal also followed the spatial organization of the underlying nanostructured topography. Hence, this indicates that vertical pillar topographies of 550 nm in diameter do not cause the disassembly of presumptive podosomes on immature hDCs, but, instead, they promote their spatial patterning. Although the definition and function of the different types of podosomes and podosomes‐like structures, such as phagocytic podosomes, that exist is often diverse,^[^ ^24^, ^27^ ^]^ we named these topography‐induced adhesive actin structures “hollow podosomes” as they are made of a rich actin structure surrounded by focal adhesion proteins around the void imposed by vertical pillar structure from which radial actin fibers extend.

### 2.2 1D Vertical Pillar Topographies Modulate the Dynamics of F‐Actin Assembly

**Figure 2:**
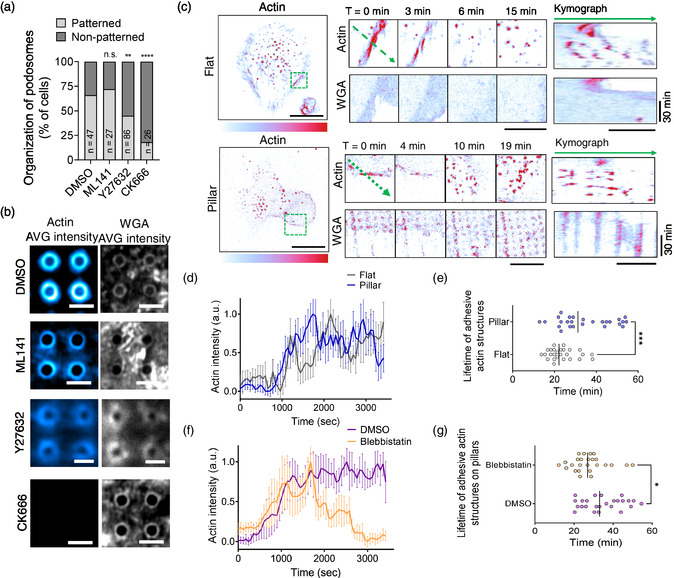
Effect of actin‐assembly factors on actin organization and dynamics in hDCs seeded on vertical pillar topographies. a) % of hDCs seeded on vertical pillar topographies showing patterned or nonpatterned podosomes on control conditions (DMSO) or after 30 min treatment with ML141 (Rac1/Cdc42 inhibitor), Y27632 (ROCK inhibitor), or CK666 (Arp2/3 complex inhibitor). Chi‐square test: ^****^ *P* < 0.0001; ^**^ *P* < 0.01; n.s. *P* > 0.05. Adjusted P values by Holm‐Šídák. Cells (*n*) analyzed from ≥ 2 biological replicates, *n*, are shown in the graph. b) Average intensity images of F‐actin organization (phalloidin, blue) and the plasma membrane morphology (WGA, gray) from *n* ≥ 7 hDCs cultured on vertical pillar topographies in control (DMSO) or in the presence of the inhibitors in a. Scale bar, 1 μm. c) Whole cell image and spinning disk snapshots from the green dashed box in the corresponding image showing the dynamics of F‐actin (spyActin‐555) and the plasma membrane (WGA Alexa 488) of hDCs cultured on flat or vertical pillar topographies (CET 19 LUT). Scale bar, 20 and 10 μm (inset). Kymograph analysis along the green dashed arrow in the corresponding image. Scale bar, 5 μm and 30 min. d) Average F‐actin intensity versus time of podosomes on hDCs cultured on flat (gray) or vertical pillar topographies (blue). Solid line represents the mean ± s.d. of *n*, number of podosomes analyzed, *n* = 10 and *n* = 10, respectively. **e)** Lifetime of podosomes on hDCs cultured on flat (gray) or vertical pillar topographies (blue). Solid line represents the mean. Podosomes analyzed (*n*) from 2 biological replicates, *n* = 29 and *n* = 29, respectively. Mann–Whitney test: ^***^ *P* < 0.001. f) Average F‐actin intensity versus time of podosomes on hDCs cultured on vertical pillar topographies in control (DMSO, magenta) or after 30 min treatment with blebbistatin (myosin‐II inhibitor, orange). Solid line represents the mean ± s.d. of *n*, number of podosomes analyzed, *n* = 10 and *n* = 10, respectively. g) Lifetime of podosomes on hDCs cultured on vertical pillar topographies in control (DMSO, magenta) or after 30 min treatment with blebbistatin (orange). Solid line represents the median. Podosomes analyzed (*n*) from 2 biological replicates, *n* = 19 and *n* = 17, respectively. Mann–Whitney test: ^*^ *P* < 0.05.

The assembly of F‐actin structures is often regulated by the Ras homology (Rho) family of small GTPases (Rho‐GTPases) through the different activation of nucleation factors. Furthermore, in monocytic cells, the formation of structures that are rich in F‐actin, such as podosomes or the phagocytic cup, depends on the Arp2/3 complex and its activation by the WASP‐family of nucleation‐promoting factors.^[^ ^27^, ^28^ ^]^ Therefore, we investigated the contribution of all these F‐actin assembly factors, RhoA and Rac1/Cdc42 as well as the Arp2/3 complex, in the patterning of podosomes on vertical pillar topographies (**Figure** **2**a and b). To this end, we seeded hDCs on vertical pillar topographies for 4 h, and we subsequently treated the cells with different F‐actin assembly inhibitors such as ML141 that inhibits Rac1/Cdc42, Y27632 that inhibits the RhoA effector Rho‐associated protein kinase (ROCK1 and 2), and CK666 that inhibits Arp2/3. Next, cells were fixed and labeled for F‐actin using phalloidin and imaged by Airyscan microscopy. We estimated the % of hDCs that presented a patterned spatial organization of hollow podosomes, as shown in Figure 1, on control conditions (DMSO) relative to cells treated with the different inhibitors aforementioned. Because RhoGTPase inhibitors would also affect the assembly of “conventional” podosomes,^[^ ^26^ ^]^ we expect that the ratio in the % of patterned versus nonpatterned podosomes would be only changed due to an additive or inhibitory effect of a given GTPase in the presence of the vertical topographies. As shown in Figure 2a and b, we did not detect any significant changes on the actin patterning of cells treated with the Rac1/Cdc42 inhibitor. However, we did observe that the ROCK inhibitor induced a significant 1.2‐fold reduction of hDCs presenting a patterning of actin structures, whereas in the presence of the Arp2/3 inhibitor, the phenotype was almost completely lost. This observation is consistent with the central function of the Arp2/3 complex in podosomes architecture.^[^ ^26^ ^]^

Noteworthy, we also observed that the plasma membrane of hDCs, labeled with WGA Alexa488, followed the topology imposed by the underlying SiO_2_ pillar topographies under all the F‐actin assembly inhibitors tested (Figure 2b). Therefore, indicating that F‐actin assembly inhibitors, and particularly of ROCK and Arp2/3 inhibitors, are not affecting the membrane curvature imposed by the underlying SiO_2_ pillar topographies. In monocyte‐derived cells, the actin cytoskeleton drives most of the cellular processes that require membrane deformations.^[^ ^17^ ^]^ However, a significant bottleneck in perturbing the architecture of the actin cytoskeleton is, often, the associated loss of the membrane morphology, thus, making it difficult to infer the involvement of cytoskeletal forces and passive processes that shape membranes.^[^ ^29^ ^]^ Thus, engineered SiO_2_ nanostructured surfaces should provide a strategy to uncouple the contribution of actin organization and membrane curvature on cytoskeleton‐driven processes in immune cells.

The results above indicate that ROCK and Arp2/3 are likely to participate in patterning hollow podosomes induced by the engineered vertical pillar topographies. ROCK is a kinase effector of RhoA, which controls the cortical actin cytoskeleton and myosin filaments.^[^ ^30^, ^31^ ^]^ To further understand actin assembly during the formation of patterned F‐actin‐rich structures, we monitored by spinning‐disk microscopy the actin dynamics on hDCs labeled with the live actin probe Spy555‐actin and seeded on either flat or SiO_2_ vertical pillar topographies of 550 nm in diameter (Figure 2c,e). Furthermore, to infer the topology effect of the underlying nanostructured surface, we labeled the plasma membrane of live hDCs with WGA Alexa 488. We found that the reorganization of cortical actin preceded the formation of micron‐sized F‐actin structures, both on flat and vertical pillar surfaces (highlighted by the green box and kymography in Figure 2c). Interestingly, we also observed that on hDCs seeded on SiO_2_ vertical pillar topographies, the formation of new hollow podosome structures respected the membrane curvature imposed by the underlying engineered surface. Thus, the surface topography might affect the dynamics of nucleation and/or stabilization of adhesive actin‐enriched structures. The quantification of the intensity of F‐actin structures over time showed that there was no apparent difference in the nucleation/initiation phase of the structures between the flat and pillar conditions (Figure 2d). However, we did observe a time delay in the intensity fluctuations of actin‐enriched structures when hDCs are seeded on pillar surfaces, possibly indicating an effect in the actin turnover within the structure. Furthermore, the lifetime of actin‐enriched structures, defined as the time between an actin structure appears and disappears at a given plasma membrane position, showed a ≈1.3‐fold increase in the pillar's array condition compared to flat surfaces. Collectively, these observations point out that vertical pillar topographies help to stabilize actin‐enriched structures, most likely by modulating its dynamics and turnover of actin within the structure.

A joint assembly factor in filament binding and force generation that is shared between cortical actin and podosomes is myosin‐II.^[^ ^30^ ^]^ While the function of myosin‐II at the cortex is essential to adapt and resist external stress and drive cell shape,^[^ ^32^ ^]^ the actin‐enriched cores of podosomes rely on myosin‐II to acquire mechanosensing properties and connect to adjacent actin cores through unbranched filaments.^[^ ^23^, ^33^ ^]^ Thus, we set out to investigate the contribution of myosin‐II in the assembly and patterning of hollow podosomes on hDCs cultured on the engineered vertical topographies and treated with the myosin‐II inhibitor blebbistatin after 4 h of cell seeding (Figure 2f,g). Next, we monitored by spinning‐disk microscopy the actin dynamics with the live actin probe Spy555‐actin, and we labeled the plasma membrane with WGA Alexa488. Under these conditions, we did not observe a significant change in the % of cells displaying patterning of actin‐enriched structures (Figure S4, Supporting Information) or in their assembly time (Figure 2f) between the control (DMSO treated) or blebbistatin‐treated hDC. However, we did observe a reduction in the lifetime of actin‐enriched structures in the presence of blebbistatin (Figure 2g). Hence, this suggests that while myosin‐II contractility might not be necessary for the assembly of patterned F‐actin‐rich structures, it will, however, assist their stabilization on vertical pillars, possibly by providing contractile forces allowing them to sustain the shape imposed by the underlying topography.

### 2.3 The Spatial Organization of Pattern‐Recognition and Opsonic Receptors is Modulated by Nanosized Vertical Topographies

**Figure 3:**
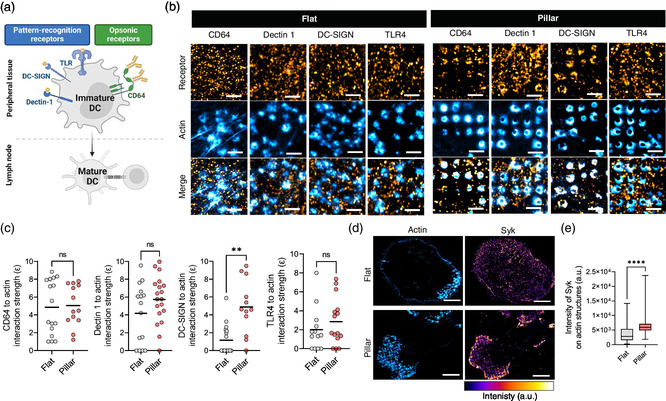
Effect of the surface topography on the spatial organization of immune receptors in hDCs. a) Schematics of different antigen‐recognition receptors, PRRs (TLR, DC‐SIGN and Dectin 1, in blue) and opsonic receptors (CD64, in green) at the surface of immature DCs at peripheral tissues. Activation of DCs upon pathogen‐mediated internalization thorough PRRs, leads to DC maturation and migration to the lymph node to mediate antigen presentation to T cells. Schematics generated by BioRender. b) Representative Airyscan images showing the endogenous organization of the receptors (orange hot LUT) CD64, Dectin 1, DC‐SIGN, and TLR4 relative to podosomes (cyan hot LUT) at the plasma membrane of immature hDCs on flat or vertical pillar topographies. Scale bar, 2 μm. c) Interaction strength (*ε*) of CD64, Dectin 1, DC‐SIGN, or TLR4 with the actin signal at the plasma membrane of immature hDCs seeded on flat (gray) or vertical pillar topographies (red). Solid line represents the mean. Cells analyzed (*n*) from 2 biological replicates, *n* = 16, *n* = 15, *n* = 17, *n* = 13 for flat and *n* = 13, *n* = 19, *n* = 12, *n* = 15 for pillar topographies, respectively. Mann–Whitney test: n.s. *P* > 0.05, ^**^ *P* < 0.01. d) Representative Airyscan images of endogenous Syk (Fire LUT) and F‐actin (cyan hot LUT) at the ventral membrane of hDCs cultured on flat or vertical pillar topographies. Scale bar, 10 μm. e) Box plot of the intensity of endogenous Syk at podosome structures of hDCs seeded on flat (gray) or vertical pillar topographies (red). Data represents 2 biological replicates. Mann–Whitney test: ^****^ *P* < 0.0001.

The surface of DCs exposes a wide variety of immune receptors providing specific functions in innate immunity.^[^ ^1^, ^28^ ^]^ This is the case of pattern recognition receptors (PRRs), which directly recognize specific molecular structures, known as pathogen‐associated molecular patterns, at the surface of external agents. Monocyte‐derived cells also co‐express opsonin receptors, which recognize adherent opsonins, and are characterized to mediate phagocytosis. PRRs can localize at protrusive podosome‐like structures in immature DCs to mediate antigen sampling.^[^ ^34^ ^]^ However, these types of podosomes display the opposite membrane morphology to that expected on vertical pillar topographies. Therefore, we asked how vertical pillar topographies might affect the spatial organization of immune receptors (**Figure** **3**a). To this end, we endogenously labeled specific PRRs and opsonin receptors and F‐actin on hDCs seeded on flat or vertical pillar topographies. hDCs were then fixed and imaged by Airyscan microscopy (Figure 3b). We focused our study on CD64, which belongs to the Fc family of phagocytic receptors (FcR) that recognize the Fc portion of opsonizing immunoglobulins, DC‐SIGN and Dectin 1, which belong to the C‐type lectin receptors (CLRs) and recognize carbohydrates on the surface of pathogens, and the toll‐like receptor 4 (TLR4), which recognizes bacterial lipopolysaccharides and plays an important role in inflammatory responses. CLRs and TLR4 belong to the group of PRRs in myeloid cells.^[^ ^1^ ^]^ We confirmed that in control (flat) conditions, actin did not accumulate with immune receptors (Figure 3b), as previously reported in resting states.^[^ ^28^ ^]^ We observed, however, that Dectin‐1 showed discrete spots positive for F‐actin and the receptor (left panel in Figure 3b). This trend was enhanced on hDCs seeded on vertical pillar topographies with a visible patterning of Dectin‐1 but also of DC‐SIGN and CD64 that was convoyed by a marked co‐localization with F‐actin‐rich structures (Figure 3b, right panel). Next, we computed the interaction strength (*ε*), as described in,^[^ ^35^ ^]^ between the actin and each receptor signal to quantitatively infer the contribution of the actin cytoskeleton on the spatial organization of immune receptors (Figure 3c). We found that TLR4 and DC‐SIGN showed a low interaction strength with F‐actin when hDCs are seeded on flat surfaces, indicating that their spatial organization is independent of the presence of actin. However, the interaction with F‐actin structures significantly increased when hDCs were cultured on vertical pillar topographies, but only for DC‐SIGN. Furthermore, we found that CD64 and Dectin 1 showed specific interaction in the presence of flat surfaces with no significant changes due to the vertical pillar topography, indicating that their spatial organization could be, in part, dictated by the actin cytoskeleton and without any interference from engineered surfaces. Collectively, these results indicate that vertical pillar topographies promote the patterning of PRR and opsonic receptors and that this organization is likely driven by a topography‐mediated process and potentiated by the association of immune receptors with F‐actin‐rich structures, in the case of CD64 and Dectin 1.

CD64 and Dectin 1 contain immunoreceptor tyrosine‐based activation motifs (ITAMs) in their cytosolic domains that are phosphorylated by tyrosine kinases and can directly recruit and activate the spleen tyrosine kinase Syk.^[^ ^21^ ^]^ Importantly, Syk is a key component in activating immune responses downstream of PRRs and opsonic receptors and a central organizer of the actin cytoskeleton in monocyte‐derived cells. Thus, we investigated the contribution of vertical pillar topographies on the spatial organization of endogenous Syk through its immunofluorescence detection in fixed hDCs previously seeded on flat or vertical pillar arrays (Figure 3d and e). We found that while endogenous Syk showed a relatively homogenous distribution at the plasma membrane interface with the substrate, it appeared accumulated to topography‐induced patterned F‐actin structures on hDCs cultured on nanostructured surfaces (Figure 3d). Furthermore, we observed a significative increase in the intensity of endogenous Syk relative to the surface of F‐actin‐rich structures in the presence of vertical pillar arrays compared to flat surfaces (Figure 3e). Hence, indicating that Syk is likely to be involved in the assembly of patterned F‐actin structures in hDCs cultured on vertical pillar topographies, in line with its known function regulating the actin cytoskeleton of monocyte‐derived cells.^[^ ^20^ ^]^

### 2.4 Membrane Shape Modulates ERK Signaling in Human Dendritic Cells

**Figure 4:**
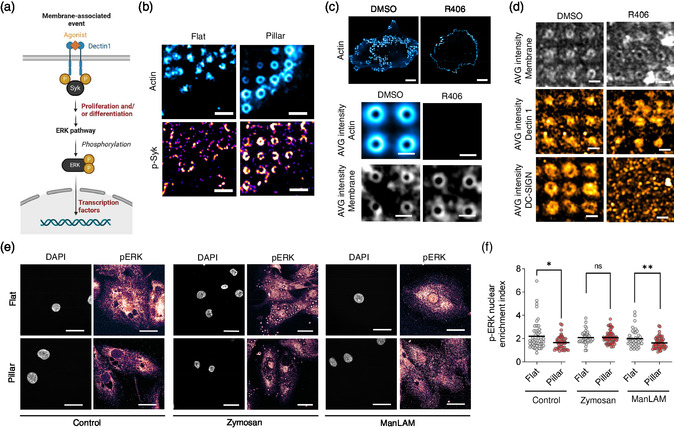
Effect of the surface topography on Syk and ERK signaling in hDCs. a) Schematics of the Syk and ERK signaling pathway. Activation of PRRs (such as Dectin 1) by agonists leads to double ITAM motif phosphorylation and binding of Syk. Syk activation can activate downstream signaling pathways, such as the ERK pathway involved in cell proliferation and differentiation through ERK phosphorylation and translocation into the nucleus to promote the activation of transcription factors, adapted from.^[^ ^21^ ^]^ Schematics generated by BioRender. b) Airyscan image showing the organization of endogenous phosphorylated Syk (pSyk, Fire LUT) relative to F‐actin (cyan hot LUT) at regions with podosomes Scale bar, 2 μm. c) Airyscan image showing the cellular organization of F‐actin (cyan hot LUT) on hDCs cultured on vertical pillar topographies and average intensity of F‐actin (cyan hot LUT) and the plasma membrane (gray) at actin‐enriched structures in control condition (DMSO) or treated with R406 (Syk inhibitor). Scale bars, 20 μm and 1 μm, respectively. d) Average intensity of the organization of the plasma membrane (gray) or the CLRs Dectin 1 and DC‐SIGN (orange hot LUT) on hDCs cultured on vertical pillar topographies in control conditions (DMSO) or treated with R406. Scale bar, 1 μm. e) Confocal images of endogenous p‐ERK (fire LUT) and the nucleus (DAPI, gray) of hDCs cultured on flat or vertical pillar topographies in control conditions (IMDM medium) or in the presence of the CLR agonists zymosan (Dectin 1 ligand) or ManLAM (DC‐SIGN ligand). Scale bar, 20 μm. f) Quantification of the pERK nuclear enrichment of hDCs cultured on flat (gray) or vertical pillar topographies (red) in control conditions (IMDM medium) or in the presence of the CLR agonists zymosan or ManLAM. Solid line represents the mean. Cells analyzed (*n*) from 2 biological replicates, *n* = 53, *n* = 42, *n* = 42 on flat and *n* = 49, *n* = 65, *n* = 59 on pillar topographies, respectively. Mann–Whitney test: n.s. *P* > 0.05, ^*^ *P* < 0.05, ^**^ *P* < 0.01. Adjusted *P* values by Holm–Šídák.

The results above suggest that Syk‐mediated signaling might be engaged on hDCs cultured on nanostructured surfaces, by promoting actin cytoskeleton reorganization and/or Syk‐mediated signal transduction downstream of ITAM motifs.^[^ ^20^, ^21^ ^]^ In this line, active Syk was shown to localize at podosomes in resting monocyte‐derived cells.^[^ ^21^ ^]^ The Syk pathway is initiated through the dual phosphorylation by SRC family kinases, leading to Syk recruitment (**Figure** **4**a). This binding promotes direct binding of partners and the downstream activation of signaling components leading to cytoskeletal changes and cellular responses such as cell proliferation, differentiation, survival or cytokine release. Hence, we analyzed by immunofluorescence the endogenous presence of phosphorylated Syk (pSyk) as an indicator of the Syk pathway activation on fixed hDCs as a function of the surface topography (Figure 4b). We observed that the signal of pSyk mirrored that of actin‐enriched structures on hDCs seeded both on flat or nanostructured vertical pillar topographies, hence evidencing a topography‐induced patterning of pSyk on the latter case. Inhibition of Syk with the selective inhibitor R406^[^ ^36^ ^]^ led to the disassembly of patterned actin‐enriched structures on hDCs seeded on vertical pillar topographies, in agreement with its reported role in actin organization and podosome assembly.^[^ ^20^ ^]^ However, Syk inhibition did not modify the membrane shape (Figure 4b). Thus, we used this feature to deduce the contribution of the membrane morphology on the spatial organization of CLRs, DC‐SIGN, and Dectin 1, as they showed an enhanced interaction strength with actin on vertical pillar topographies (Figure 3b and c), and, in the case of Dectin‐1 it also holds an ITAM motif that binds Syk.^[^ ^37^ ^]^ As shown in Figure 4d, we found that inhibition of Syk on hDCs seeded on vertical pillar topographies was convoyed by a marked loss of DC‐SIGN patterning in contrast to Dectin 1 that, instead, kept a patterned organization. Hence, suggesting that the topography‐mediated patterning of CLRs is coupled to the Syk pathway in a receptor‐specific manner: while the spatial organization of DC‐SIGN depends on active Syk, Dectin‐1 patterning is likely to take place upstream of Syk and could be, eventually, linked to the membrane morphology.

The SyK signaling pathway is regulated by several feedback mechanisms, and the direct binding of Syk with partners and downstream intermediates leads to cellular responses such as cell proliferation and/or differentiation, which is mediated by the translocation of phosphorylated ERK into the nucleus and the activation of specific transcription factors.^[^ ^21^ ^]^ Hence, we set out to inquire about the effect of vertical pillar topographies in the translocation of phosphorylated ERK (pERK) into the nucleus of hDCs. To this end, we immunolabeled pERK on fixed hDCs that were previously seeded for 4 h on either flat or vertical pillar topographies and imaged by confocal microscopy (Figure 4e and f). Furthermore, we labeled the nucleus of hDCs with DAPI for further segmentation and image analysis. We found that seeding of hDCs on vertical pillar topographies is convoyed by a significant decrease of pERK enrichment at the nucleus, compared to control conditions (i.e., on flat surfaces) (Figure 4f). We observed the same trend in the case of ERK (Figure S5, Supporting Information). Next, we stimulated hDCs with specific CLR agonists. In particular, we incubated hDCs for 1 h with zymosan, which activates Dectin‐1, and ManLAM, which activates DC‐SIGN, then cells were fixed and stained for pERK and DAPI, and analyzed (Figure 4e and f). We observed that stimulation of hDCs with zymosan rescued the translocation of pERK at the nucleus in cells seeded on vertical pillar topographies, hence showing an equivalent pERK enrichment in the nucleus of cells cultured on flat or nanostructured surfaces. This was not the case with ManLAM, where pERK translocation in the nucleus remained unaffected in the presence of the agonist and thus, appeared still significantly lower in hDCs cultured on vertical pillar topographies. Collectively, these results indicate that nanostructured surfaces can modulate cellular responses in hDCs, particularly targeting the ERK pathway and that this effect is maintained under the presence of specific agonists such as ManLAM. Hence, the effect of pillar topographies in ERK pathway modulation in hDCs might arise from the spatial constraint imposed by the topography and/or the accumulation of CLRs, such as DC‐SIGN, and pSyk at patterned actin‐enriched structures. Either situation might convoy changes in the receptor mobility and a steric hindrance leading to a different association of cytosolic receptor domains and pSyk with proximal downstream partners and signaling intermediates and/or modify the amplification of the signaling cascade. In this line, actin‐enriched nano‐deformations in cancer cells were shown to lead to partial inhibition of IFNγ‐induced JAK/STAT signaling by locally accumulating the interferon‐γ receptor.^[^ ^13^ ^]^

Our study showed an attenuated response to immune receptor agonists upon surface‐topography‐induced preclustering of receptors, for instance, in the case of DC‐SIGN. The C‐type lectin DC‐SIGN, which is highly expressed at the surface of DCs, mediates the entry of several pathogens, including viruses like HIV‐1.^[^ ^38^, ^39^ ^]^ DC‐SIGN recognizes mannosylated and fucosylated ligands, such as ManLAM or the HIV gp120 envelope. In our study, we used ManLAM glycans. However, soluble ligands might not recapitulate the local effect of pathogen binding and receptor engagement. Thus, how surface topography influences pathogen recognition is a question that should be further explored, for instance, in the context of HIV‐1 infection. DC‐SIGN was shown to have a role in viral antigen presentation while also contributing to enhanced transmission of HIV‐1 infection.^[^ ^40^ ^]^ Thus, vertical pillar topographies could also be exploited to address how the degree of receptor preclustering might compensate for the transmission of infection to T‐cells.

Finally, our findings showing topography‐induced changes in immune receptors indicate that they could be used to selectively modulate specific immune cell functions for biomedical applications. Our work shows that engineered surface topographies will likely attenuate the host's immune response. Consequently, they could be used, for instance, to improve the host tolerance to medical device implants and immunotherapy applications to achieve and/or modulate a precise molecules/antigen delivery. This application has been shown, for instance, in murine models where biomaterials of defined chemistries and topologies on murine DCs were tailored to achieve desired levels of immune cell activation for specific applications.^[^ ^41^, ^42^ ^]^ However, these applications must be explored further in the context of human DCs. Another study, this time using tlr‐/‐ mice showed that TLRs could act as sensors of specifically designed biomaterials leading to enhanced expression of activation markers and proinflammatory cytokines, hence conferring the ability to murine DCs to activate antigen‐specific T cells.^[^ ^43^ ^]^ Therefore, it would be interesting to investigate further the suitability of engineered surface topographies to modulate the secretion of specific cytokines by hDCs, such as proinflammatory cytokines or chemokines, signaling events, and antigen routing/processing into specific intracellular MHC‐II compartments, and the subsequent activation of antigen‐specific T‐cells.

## 3 Conclusion

A central function of DCs is to support immune surveillance against external agents such as pathogens or aberrant cells.^[^ ^1^ ^]^ As they patrol throughout different tissues in the human body, DCs mus*t* test and sense the microenvironment and integrate different biochemical and physical cues to engage a proper cellular response.^[^ ^3^ ^]^ To support this essential function, the outmost barrier of DCs, the plasma membrane, is likely to act as a multifunctional interface interconnecting: i) receptors laterally organized that will transduce signals across the membrane and activate downstream signaling molecules; ii) support morphological changes; and iii) the coupling with cytoskeleton structures.^[^ ^17^, ^21^, ^26^, ^30^ ^]^ In this work, we set out to directly manipulate the membrane morphology of hDCs to investigate how these changes transduce to specific cellular responses. We showed that by modulating the environment topography with SiO_2_ vertical pillars, it is possible to impact the cellular response of DCs through changes in the spatial organization of the actin‐enriched adhesive structures, namely hollow podosomes, immune receptors, such as CD64, DC‐SIGN, and Dectin 1, and associated signaling molecules such as Syk and ERK1/2. DC‐SIGN and Dectin 1 fulfill central immune functions through, pathogen uptake and the expression of specific cytokines, contributing to adaptative responses such as antigen presentation or T‐cell differentiation.^[^ ^44^ ^]^ We show that by imposing changes in defined immune receptors patterning and their association with the actin cytoskeleton, the DC response can be modulated from the plasma membrane remodeling. In this work, we explored the role of ERK signaling, which regulates different essential pathways shaping the fate and response of cells, including inflammatory cytokine expression and cell metabolism, survival, proliferation, and migration.^[^ ^45^ ^]^ Collectively, this work shows that bioengineering of microenvironments allowing the manipulation of the local membrane morphology of immune cells could be used to investigate fundamental biological processes but also as a strategy to ex vivo modulate their cellular fate, for instance, in immunotherapies based on the delivery of ex vivo expanded DCs.^[^ ^46^ ^]^

## 4 Experimental Section

4.1

4.1.1

##### Reagents and Antibodies

The following antibodies were used in this study: Paxillin polyclonal rabbit was from Genetix (GTX125891‐S). Monoclonal Rabbit avb3 integrin (ZRB1190) was from Merck Sigma‐Aldrich. Mouse monoclonal anti‐Dectin1/CLEC7A (Cat. No. MAB1859) was from R&D systems. Mouse monoclonal p‐ERK (sc 7383), rabbit polyclonal ERK (sc 94), and mouse monoclonal DC‐SIGN (MR‐1) (sc 59 157) were from Santa Cruz Biotechnology. Mouse monoclonal TLR4 (Cat. No. 66 350) was from protein technology. Secondary antibodies were from Jackson ImmunoResearch Laboratories Inc.: donkey anti‐Mouse IgG (H + L) conjugated to Cy3 (Cat. No: 715‐165‐150) and Alexa Fluor 488 (Cat. No: 715‐545‐150). Donkey anti‐Rabbit IgG (H + L) conjugated to Cy3 (Cat. No. 715‐165‐152) and Alexa Fluor 488 (Cat. No: 611‐545‐215). SPY555‐Actin from Spiro‐chrome (cat. SC202) was used for live‐cell staining of F‐actin. Phalloidin‐Atto 647N or Atto 390 from Sigma‐Aldrich was used to stain F‐actin in fixed cells. Plasma membrane staining was performed with WGA Alexa 488 from Invitrogen.

Dimethyl sulfoxide (DMSO) extracellular signal‐regulated kinases (CAS. 67‐68‐5) was from Sigma. The following inhibitors were used: R406 (5 μM, Cat. No: SE‐S2194) and Y27632 (10 μM, Cat. No. S1049) were from Selleckchem. ML141 (22.5 μM, SML0407) (CAS. 71 203‐35‐5), blebbistatin (50 nM, CAS: 856 925‐71‐8), CK666 (0.1 mM, CAT. No: 442 633‐00‐3) were from Merck Sigma‐Aldrich. Fibronectin bovine plasma (0.1 mg mL^−1^ Cat. No: F1141) and zymosan (0.1 mg mL^−1^, CAS. No. 58 856‐93‐2) were purchased from Merck Sigma‐Aldrich. ManLAM was purified from *Mycobacterium bovis* BCG as previously reported.^[^ ^47^ ^]^

##### Preparation of Ex Vivo Human Monocyte‐Derived Dendritic Cells

Human monocytes from buffy coats were obtained according to institutional guidelines of the ethical committee of the CNRS and Établissement Français du Sang (EFS). After Ficoll gradient on peripheral blood mononuclear cells, monocytes were isolated using CD14 MicroBeads (Miltenyi Biotec, Bergisch Gladbach, Germany). Usual purity was >95% CD14^+^. Human MoDC were generated by incubating purified monocytes in IMDM‐GlutaMAX^TM^ (Gibco, Thermo Fisher Scientific, Waltham, MA USA) supplemented with 10% FCS, 100 IU mL^−1^ penicillin, 100 μg mL^−1^ streptomycin, 10 mM Hepes, 1% nonessential amino acids, 1 mM sodium pyruvate (all from Merck KGaA, Darmstadt, Germany), and cytokines GM‐CSF (500 IU mL^−1^) and IL‐4 (500 IU mL^−1^) (both from Miltenyi Biotec, Bergisch Gladbach, Germany). The first day, 50 μM of 2‐mercaptoéthanol was added to limit high levels of free radicals. The obtained immature MoDC were harvested at day 5–6 and phenotyped by flow cytometry before experimental use.

##### Synthesis of Silica Sol–Gel Thin Films and Nanostructuration

The procedure of SiO_2_ micro and nano‐structuration allows to obtain periodic patterns with a controlled shape, diameter, and periodicity over a large surface (≈cm^2^) on conventional high‐quality borosilicate glass coverslips.^[^ ^12^, ^22^ ^]^ Briefly, a solution was prepared using 23.26 g absolute ethanol, 1.5 g HCl (37%), and 4.22 g tetraethyl orthosilicate (TEOS), and the solution was stirred for 16 h. All reagents were from Sigma‐Aldrich. The sol–gel thin films were coated by dip‐coating method. The process used ambient temperature (25 °C) and relative humidity at 45%–50%, and the thickness of the film was controlled by a withdrawal speed of 300 nm min^−1^ in order to adjust the final thickness to 200 nm. Then, silica layer was consolidated with a thermal treatment of 5 min at 450 °C under air atmosphere. The multilayer gel films were obtained by repeating two times the process of monolayer preparation on the same substrate. After the second silicate layer coating the substrate, the film was imprinted with a (PDMS mold containing the different vertical pillar sizes and pitch. Finally, these nanostructured thin films were consolidated with a thermal treatment of 10 min at 450 °C under an air atmosphere.

Structural characterization of the different SiO_2_ vertical pillar topographies used in this study is displayed in Table S1, Supporting Information, and was carried out by atomic force microscopy and FEG‐scanning electron microscope model Su‐70 Hitachi, equipped with an energy dispersive X‐ray (EDX) detector X‐max 50 mm^2^ from Oxford Instruments.

##### Immunofluorescence

hDCs were seeded on fibronectin‐coated SiO_2_ nanostructured surfaces, left to adhere for 4 h. Before fixation, cells were incubated 6 min with WGA Alexa 488 (1/200 dilution). Cells were then fixed in 3.2% w/v paraformaldehyde and permeabilized in 0.1% v/v Triton X‐100 following classical methods. Cells were incubated with primary antibodies for 45 min. Subsequently, phalloidin and secondary antibodies were incubated for 45 min. Finally, coverslips were mounted with a Mowiol 4‐88 mounting medium (Polysciences, Inc.). Montage was allowed to solidify in the dark for 48 h before microscope acquisitions.

##### Airyscan Confocal Microscopy

Images were acquired on a Zeiss LSM980 Airyscan confocal Microscope (MRI facility, Montpellier). Excitation sources were 405 nm diode laser, an argon laser for 488 and 514 nm, and a helium/neon laser for 633 nm. Acquisitions were performed on a 63X/1.4 objective. Multidimensional acquisitions were acquired via an Airyscan detector (32‐channel GaAsP photomultiplier tube array detector). 3D images were acquired by fixing a 0.25 μm z‐step.

##### Spinning Disk Microscopy of Living Cells

Live cell imaging of hDC cells seeded on arrays of vertical nanopillar with *D* ≈550 nm was performed using a spinning disk confocal microscope (Nikon TI Andor CSU‐X1 equipped with a Yokogawa spinning disk unit (Andor); MRI facility, Montpellier) equipped with a 488 laser beam (60 mW) and a 633 laser beam (mW). Acquisitions were performed with a 100X/1.45 objective. During imaging, cells were maintained at 37 °C with 5% CO_2_ in an onstage incubator (Okolab). Movies were recorded first with a dichroic mirror (488 nm) and a green fluorescence protein (GFP) emission filter (525–530 nm) and second with dichroic mirror (638 nm) and a Cy5 emission filter (685–740 nm). Samples were exposed to laser light for 100 ms every 2 s for 5 min, and images were acquired using an EMCCD iXon897 (Andor) camera. A 0.25 μm z‐step was used to cover all of the pillar's length in every z‐stack acquisition. Pictures were acquired with a time frame of 60 sec during 1 h.

##### STED Imaging

Images were acquired on an Abberior STED super‐resolution microscope (MRI facility, Montpellier). Excitation sources used were as follows: 405, 488, 561, and 640 nm lasers. Acquisitions were performed on a 60X/1.2 W objective. Multidimensional acquisitions were acquired via 4 high quantum efficiency Avalanche photodiodes detector.

##### Spatial Statistical Analysis

Spatial statistical analyses were made with R software and the following R packages, as previously described in.^[^ ^48^ ^]^

Coordinates of actin‐enriched structures have been manually detected based on fluorescent images using the multipoint function in Image J.^[^ ^49^ ^]^ Micron‐sized actin‐rich structures were defined as individual structures with an average diameter of 0.5–1 μm in the control condition (i.e., flat surface) and for the pillar condition (i.e., vertical pillars of 550 nm diameter) when a pillar is surrounded by the actin signal > 50% of its diameter. Moreover, a cell mask was generated with a thresholding based on fluorescent images and defined the observation window Ω.

For a set of *n* micron‐sized actin structure coordinates $X=\left\{x_{1},\;x_{2},\ldots ,x_{n}\right\}\;$ observed in an area |Ω|, The Ripley's K function^[^ ^50^, ^51^ ^]^ is defined as

(1) $$K\left(r\right)=\frac{\left|\Omega \right|}{n\left(n-1\right)}\sum_{i=1}^{n}\sum_{j\neq i}1_{\left|x_{i}-x_{j}\right|\leq r}$$

This function quantifies the average number of micron‐sized actin structures in a disk of radius *r* centered on one micrometric actin structure. In case of CSR, i.e., uniformly and independent distribution of points, the estimator of this function should fluctuate around $\pi r^{2}$. Therefore, we subtracted $\pi r^{2}$ from K(r). The spatial Ripley's K function has been computed using spatstat^[^ ^52^ ^]^ function Kest() with the best edge correction possible. Ripley's K function was computed between 0 and 15 μm to avoid edge effects and because our interest is to evaluate the effect at the scale of pillar spacing. Ripley's K functions plotted are an average of a population of Ripley's K functions evaluated for single cells (± SEM). A permutation test (with 999 permutations) based on a Studentized distance is used to compare populations thanks to the sptatstat^[^ ^52^ ^]^ function studpermu.test().^[^ ^53^ ^]^

Moreover, a multivariate form of Ripley's K function has been used to quantify the interaction between micron‐sized actin structures (AS) and pillars. This cross Ripley's K function^[^ ^51^ ^]^ is defined as

(2) $$K_{\text{AS,pillar}}\left(r\right)=\frac{\left|\Omega \right|}{n_{\text{AS}}n_{\text{pillar}}}\sum_{i=1}^{n_{\text{AS}}}\sum_{j=1}^{n_{\text{pillar}}}1_{\left|x_{i}-y_{j}\right|\leq r}$$

With $n_{\text{AS}}$ and $n_{\text{pillar}}$ the respective number of observed micron‐sized actin structures and pillars in the observed area |Ω|; and $X=\left\{x_{1},\;x_{2},\;\ldots ,x_{n_{\text{AS}}}\right\}$ and $Y=\left\{y_{1},y_{2},\ldots ,y_{n_{\text{pillar}}}\right\}$ the respective coordinates of micrometric actin structures and pillars. Noteworthy that this cross function is symmetric, i.e., $K_{\text{AS},\text{pillar}}\left(r\right)=K_{\text{pillar},\text{AS}}\left(r\right)$, but its estimators may not be exactly symmetric due to edge corrections. Here, the reported cross Ripley's K function is actually the average of the two. Similarly, to the univariate form, we retract $\pi r^{2}$ because it is the expected value in case of independence between *X* and *Y*. The cross‐K function gives information on the interactions, a $K_{\text{AS},\text{pillar}}\left(r\right)>0$ indicates attraction between the two point types at a distance *r*, whereas $K_{\text{AS},\text{pillar}}\left(r\right)<0\;$ indicates repulsion. The cross Ripley's K function has been computed using spatstat^[^ ^52^ ^]^ function Kcross() with the best edge correction possible.

Finally, Monte‐Carlo simulations were used to generate CSR envelopes as in ref.[48] Because observed Ripley's K function is averaged over a population of cells; CSR simulations were run in the same way. For each cell, a Monte Carlo CSR simulation was run using the observed cell mask and the observed number of points. The resulting Ripley's K functions are averaged over the cells. This procedure was repeated a high number of times (n=100). The CSR envelope contains 95% of these averaged (over the cells) Ripley's K functions, and the solid line is the average (over the simulations). This procedure controls accurately CSR deviation to $\pi r^{2}$ that could be due to border effects. Moreover, this procedure takes in consideration the effect of the pillars. Indeed, Ripley's K function is computed ignoring pillars (i.e., the observation window Ω is a simply connected set), but CSR simulations were run over a cell mask with holes for pillars (i.e., not a simply connected set). The holes in the mask has been generated using the known diameter of the pillar and their spacing, in addition of the measured coordinates of 4 of them and their directions (hence the pillar grid can be extended). CSR envelopes for the cross Ripley's K function have been generated in the same way (keeping the grid of pillars constants but running Monte‐Carlo simulation for micron‐sized actin structure localization). Cell masks, micron‐sized actin structures coordinates, and the codes are available at Github: https://github.com/Hugo‐Lachuer/Rathar.et.al.2024.

##### pERK and ERK Analysis

Analysis of endogenous pERK or ERK enrichment at the nucleus was performed on hDCs cells seeded on either flat or vertical pillar topographies during 4 h. To facilitate the visualization of pERK or ERK accumulation at the nucleus cells were incubated with 0.05 μM Leptomycin B (LMB), an inhibitor of nuclear export. Then, cells were fixed and immunofluorescence using selective antibodies for either pERK or ERK and DAPI (nucleus staining) and imaged by confocal microscopy.

Confocal images of endogenous pERK and ERK and nucleus staining were analyzed using a semi‐manual ImageJ macro. First, a mask of the cell nucleus was generated with the DAPI channel by thresholding (mask_nucleus_). Then the cell was manually detected with a thresholding to define the cytoplasm area (mask_cytoplasm_). To determine the pERK nuclear enrichment (E_pERK.nuclear_) on hDCs cultured on flat or vertical pillar arrays the following formula was applied: E_pERK.nuclear_ = MGV_pERK nuclear_/MGV_pERK cytoplasm_, where MG_pERK nuclear_ corresponds to the mean gray value of the pERK signal at mask_nucleus_ and MGV_pERK cytoplasm_ corresponds to the mean gray value of the of the pERK signal at the cytoplasm without the nucleus (mask_cytoplasm_ – mask_nucleus_). The same analysis was applied in the case of ERK.

##### Data Analysis, Representation, and Statistical Tests

Image processing and quantification were performed using ImageJ.^[^ ^49^ ^]^ 2D ACF analysis was performed using the open‐source software Gwyddion.^[^ ^54^ ^]^ Data representation was performed using Origin and Prism GraphPad software. Statistical analysis was performed using the Mann–Whitney two‐tailed test or a chi‐square test for discrete statistics between proportions for the different conditions using the Prism GraphPad software. In all statistical tests, the levels of significance were defined as ^ *^ *P* < 0.05, ^**^ *P* < 0.01, ^***^ *P* < 0.001, and ^****^ *P* < 0.0001.

## Conflict of Interest

The authors declare no conflict of interest.

## Author Contributions

**Raïssa Rathar, Adrian Carretero‐Genevrier** and **Laura Picas**: Conceptualization; **Raïssa Rathar, David Sanchez‐Fuentes, Hugo Lachuer, Valentin Meire, Aude Boulay, Rudy Desgarceaux, Fabien P. Blanchet and Laura Picas**: Methodology and/or data analysis; **Adrian Carretero‐Genevrier and Laura Picas**: Supervision; **Adrian Carretero‐Genevrier and Laura Picas**: Funding acquisition; **Raïssa Rathar, Laura Picas and Adrian Carretero‐Genevrier**: Writing with inputs from all authors.

## Supporting information

Supplementary Material

## Data Availability

The data that support the findings of this study are available in the supplementary material of this article.

## References

[1] J. S. Marshall , R. Warrington , W. Watson , H. L. Kim , Allergy, Asthma, Clin. Immunol. 2018, 14, 49. 30263032 10.1186/s13223-018-0278-1 PMC6156898

[2] R. N. Germain , E. A. Robey , M. D. Cahalan , Science 2012, 336, 1676. 22745423 10.1126/science.1221063 PMC3405774

[3] D. Vesperini , G. Montalvo , B. Qu , F. Lautenschläger , Biophys. Rev. 2021, 13, 185. 34290841 10.1007/s12551-021-00787-9 PMC8285443

[4] A. de la Zerda , M. J. Kratochvil , N. A. Suhar , S. C. Heilshorn , APL Bioeng. 2018, 2, 021501. 31069295 10.1063/1.5006599 PMC6324202

[5] S. Adutler‐Lieber , I. Zaretsky , I. Platzman , J. Deeg , N. Friedman , J. P. Spatz , B. Geiger , J. Autoimmun. 2014, 54, 100. 24951031 10.1016/j.jaut.2014.05.003

[6] J. Deeg , M. Axmann , J. Matic , A. Liapis , D. Depoil , J. Afrose , S. Curado , M. L. Dustin , J. P. Spatz , Nano Lett. 2013, 13, 5619. 24117051 10.1021/nl403266t PMC3828117

[7] K. Shen , V. K. Thomas , M. L. Dustin , L. C. Kam , Proc. Natl. Acad. Sci. U. S. A. 2008, 105, 7791. 18505845 10.1073/pnas.0710295105 PMC2409411

[8] R. Basu , B. M. Whitlock , J. Husson , A. Le Floc'h , W. Jin , A. Oyler‐Yaniv , F. Dotiwala , G. Giannone , C. Hivroz , N. Biais , J. Lieberman , L. C. Kam , M. Huse , Cell 2016, 165, 100. 26924577 10.1016/j.cell.2016.01.021 PMC4808403

[9] K. T. Bashour , A. Gondarenko , H. Chen , K. Shen , X. Liu , M. Huse , J. C. Hone , L. C. Kam , Proc. Natl. Acad. Sci. U. S. A. 2014, 111, 2241. 24469820 10.1073/pnas.1315606111 PMC3926067

[10] H.‐Y. Lou , W. Zhao , X. Li , L. Duan , A. Powers , M. Akamatsu , F. Santoro , A. F. McGuire , Y. Cui , D. G. Drubin , B. Cui , Proc. Natl. Acad. Sci. U. S. A. 2019, 116, 23143. 31591250 10.1073/pnas.1910166116 PMC6859365

[11] M. Galic , S. Jeong , F.‐C. Tsai , L.‐M. Joubert , Y. I. Wu , K. M. Hahn , Y. Cui , T. Meyer , Nat. Cell Biol. 2012, 14, 874. 22750946 10.1038/ncb2533 PMC3519285

[12] T. Sansen , D. Sanchez‐Fuentes , R. Rathar , A. Colom‐Diego , F. El Alaoui , J. Viaud , M. Macchione , S. de Rossi , S. Matile , R. Gaudin , V. Bäcker , A. Carretero‐Genevrier , L. Picas , ACS Appl. Mater. Interfaces 2020, 12, 29000. 32464046 10.1021/acsami.0c05432

[13] B. Ledoux , N. Zanin , J. Yang , V. Mercier , C. Coster , C. Dupont‐Gillain , D. Alsteens , P. Morsomme , H.‐F. Renard , Sci. Adv. 2023, 9, eade1660. 38091386 10.1126/sciadv.ade1660 PMC10848735

[14] L. Hanson , W. Zhao , H.‐Y. Lou , Z. C. Lin , S. W. Lee , P. Chowdary , Y. Cui , B. Cui , Nat. Nanotechnol. 2015, 10, 554. 25984833 10.1038/nnano.2015.88 PMC5108456

[15] C. Chiappini , J. O. Martinez , E. De Rosa , C. S. Almeida , E. Tasciotti , M. M. Stevens , ACS Nano 2015, 9, 5500. 25858596 10.1021/acsnano.5b01490 PMC4733661

[16] W. Zhao , L. Hanson , H.‐Y. Lou , M. Akamatsu , P. D. Chowdary , F. Santoro , J. R. Marks , A. Grassart , D. G. Drubin , Y. Cui , B. Cui , Nat. Nanotechnol. 2017, 12, 750. 28581510 10.1038/nnano.2017.98 PMC5544585

[17] H. D. Moreau , A.‐M. Lennon‐Duménil , P. Pierobon , J. Cell Sci. 2020, 133, jcs244806. 32122988 10.1242/jcs.244806

[18] K. Jaqaman , H. Kuwata , N. Touret , R. Collins , W. S. Trimble , G. Danuser , S. Grinstein , Cell 2011, 146, 593. 21854984 10.1016/j.cell.2011.06.049 PMC3160624

[19] C. W. Cairo , R. Mirchev , D. E. Golan , Immunity 2006, 25, 297. 16901728 10.1016/j.immuni.2006.06.012

[20] V. Jaumouillé , Y. Farkash , K. Jaqaman , R. Das , C. A. Lowell , S. Grinstein , Dev. Cell 2014, 29, 534. 24914558 10.1016/j.devcel.2014.04.031 PMC4083245

[21] A. Mócsai , J. Ruland , V. L. J. Tybulewicz , Nat. Rev. Immunol. 2010, 10, 387. 20467426 10.1038/nri2765 PMC4782221

[22] F. El Alaoui , I. Casuso , D. Sanchez‐Fuentes , C. Arpin‐Andre , R. Rathar , V. Baecker , A. Castro , T. Lorca , J. Viaud , S. Vassilopoulos , A. Carretero‐Genevrier , L. Picas , eLife 2022, 11, e73156. 35044298 10.7554/eLife.73156 PMC8798043

[23] M. Portes , T. Mangeat , N. Escallier , O. Dufrancais , B. Raynaud‐Messina , C. Thibault , I. Maridonneau‐Parini , C. Vérollet , R. Poincloux , eLife 2022, 11, e75610. 35727134 10.7554/eLife.75610 PMC9255968

[24] J. C. Herron , S. Hu , T. Watanabe , A. T. Nogueira , B. Liu , M. E. Kern , J. Aaron , A. Taylor , M. Pablo , T.‐L. Chew , T. C. Elston , K. M. Hahn , Nat. Commun. 2022, 13, 4363. 35896550 10.1038/s41467-022-32038-0 PMC9329332

[25] K. Van Den Dries , S. Linder , I. Maridonneau‐Parini , R. Poincloux , J. Cell Sci. 2019, 132, jcs236828. 31836688 10.1242/jcs.236828

[26] S. Linder , P. Cervero , R. Eddy , J. Condeelis , Nat. Rev. Mol. Cell Biol. 2023, 24, 86. 36104625 10.1038/s41580-022-00530-6

[27] S. Linder , B. Barcelona , Eur. J. Cell Biol. 2023, 102, 151356. 37625234 10.1016/j.ejcb.2023.151356

[28] S. A. Freeman , S. Grinstein , Immunol. Rev. 2014, 262, 193. 25319336 10.1111/imr.12212

[29] N. S. Gov , Phil. Trans. R. Soc. B 2018, 373, 20170115.29632267

[30] K. Rottner , J. Faix , S. Bogdan , S. Linder , E. Kerkhoff , J. Cell Sci. 2017, 130, 3427. 29032357 10.1242/jcs.206433

[31] M. P. Taylor , O. O. Koyuncu , L. W. Enquist , Nat. Rev. Microbiol. 2011, 9, 427. 21522191 10.1038/nrmicro2574 PMC3229036

[32] P. Sens , J. Plastino , J. Phys.: Condens. Matter 2015, 27, 273103. 26061624 10.1088/0953-8984/27/27/273103

[33] A. Labernadie , A. Bouissou , P. Delobelle , S. Balor , R. Voituriez , A. Proag , I. Fourquaux , C. Thibault , C. Vieu , R. Poincloux , G. M. Charrière , I. Maridonneau‐Parini , Nat. Commun. 2014, 5, 5343. 25385672 10.1038/ncomms6343

[34] M. Baranov , M. Ter Beest , I. Reinieren‐Beeren , A. Cambi , C. G. Figdor , G. Van Den Bogaart , J. Cell Sci. 2014, 12, 1052. 10.1242/jcs.141226 PMC4054684 24424029

[35] A. Shivanandan , A. Radenovic , I. F. Sbalzarini , BMC Bioinf. 2013, 14, 349. 10.1186/1471-2105-14-349 PMC4219334 24299066

[36] A. Naldi , R. M. Larive , U. Czerwinska , S. Urbach , P. Montcourrier , C. Roy , J. Solassol , G. Freiss , P. J. Coopman , O. Radulescu , PLOS Comput. Biol. 2017, 13, e1005432. 28306714 10.1371/journal.pcbi.1005432 PMC5376343

[37] I. M. Dambuza , G. D. Brown , Curr. Opin. Immunol. 2015, 32, 21. 25553393 10.1016/j.coi.2014.12.002 PMC4589735

[38] A. Cambi , F. De Lange , N. M. Van Maarseveen , M. Nijhuis , B. Joosten , E. M. H. P. Van Dijk , B. I. De Bakker , J. A. M. Fransen , P. H. M. Bovee‐Geurts , F. N. Van Leeuwen , N. F. Van Hulst , C. G. Figdor , J. Cell Biol. 2004, 164, 145. 14709546 10.1083/jcb.200306112 PMC2171967

[39] P.‐Y. Lozach , L. Burleigh , I. Staropoli , A. Amara , Glycovirol. Protoc. 2007, 379, 51. 10.1007/978-1-59745-393-6_4 PMC7122727 17502670

[40] Y. van Kooyk , T. B. H. Geijtenbeek , Nat. Rev. Immunol. 2003, 3, 697. 12949494 10.1038/nri1182

[41] L. K. Petersen , A. E. Ramer‐Tait , S. R. Broderick , C.‐S. Kong , B. D. Ulery , K. Rajan , M. J. Wannemuehler , B. Narasimhan , Biomaterials 2011, 32, 6815. 21703679 10.1016/j.biomaterials.2011.05.063

[42] S. P. Shankar , T. A. Petrie , A. J. García , J. E. Babensee , J. Biomed. Mater. Res., Part A 2010, 92A, 1487. 10.1002/jbm.a.32487 PMC10515974 19425048

[43] B. Shokouhi , C. Coban , V. Hasirci , E. Aydin , A. Dhanasingh , N. Shi , S. Koyama , S. Akira , M. Zenke , A. S. Sechi , Biomaterials 2010, 31, 5759. 20452017 10.1016/j.biomaterials.2010.04.015

[44] T. B. H. Geijtenbeek , S. I. Gringhuis , Nat. Rev. Immunol. 2009, 9, 465. 19521399 10.1038/nri2569 PMC7097056

[45] R. M. Lucas , L. Luo , J. L. Stow , Biochem. Soc. Trans. 2022, 50, 1341. 36281999 10.1042/BST20220271 PMC9704528

[46] J. Weiden , J. Tel , C. G. Figdor , Nat. Rev. Immunol. 2018, 18, 212. 28853444 10.1038/nri.2017.89

[47] L. Papin , M. Lehmann , J. Lagisquet , G. Maarifi , V. Robert‐Hebmann , C. Mariller , Y. Guerardel , L. Espert , V. Haucke , F. P. Blanchet , IJMS 2023, 24, 9008. 37240354 10.3390/ijms24109008 PMC10219569

[48] H. Lachuer , L. Le , S. Lévêque‐Fort , B. Goud , K. Schauer , Proc. Natl. Acad. Sci. 2023, 120, e2207425120. 36800388 10.1073/pnas.2207425120 PMC9974462

[49] C. A. Schneider , W. S. Rasband , K. W. Eliceiri , Nat. Methods 2012, 9, 671. 22930834 10.1038/nmeth.2089 PMC5554542

[50] B. D. Ripley , J. Appl. Probab. 1976, 13, 255.

[51] P. M. Dixon , in Wiley StatsRef: Statistics Reference Online, John Wiley & Sons, Ltd, Hoboken, Nueva Jersey, Estados Unidos 2014.

[52] A. Baddeley , E. Rubak , R. Turner , in Spatial Point Patterns: Methodology and Applications with R, Chapman and Hall/CRC Press, London 2015.

[53] U. Hahn , J. Am. Stat. Assoc. 2012, 107, 754.

[54] D. Nečas , P. Klapetek , Open Phys. 2012, 10, 181.

