# Supplementary material for: Tuning the Immune Cell Response through Surface Nanotopography Engineering
Source: Small Sci. 2024 Jul 21;4(9):2400227. doi: 10.1002/smsc.202400227 (PMC11935051; doi:10.1002/smsc.202400227)
Supplement: Supplementary file 1 — Supplementary Material [file SMSC-4-2400227-s001.pdf]

Supporting Information

Tuning the immune cell response through surface nanotopography engineering

Raïssa Rathar, David Sanchez-Fuentes, Hugo Lachuer, Valentin Meire, Aude Boulay, Rudy Desgarceaux, Fabien P. Blanchet, Adrian Carretero-Genevrier, Laura Picas*.*

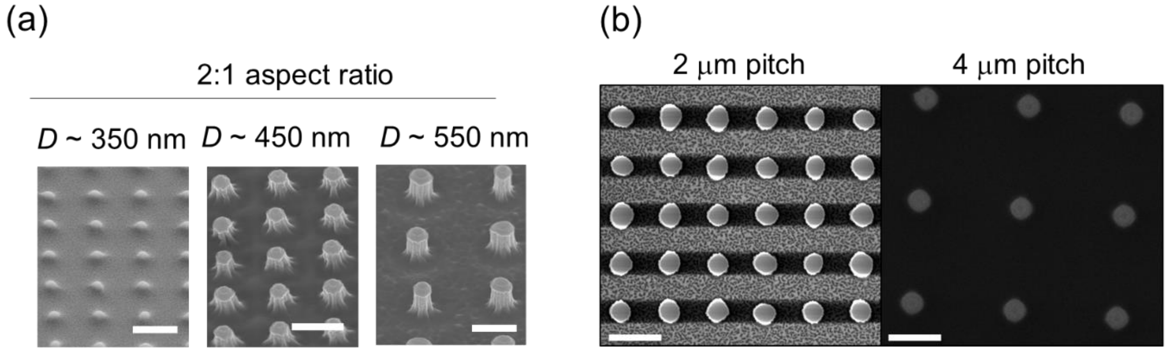

Figure S1. Scanning Electron Microscopy images of the different SiO_2 nano-structured surfaces engineered by soft-NIL on high-quality borosilicate glass coverslips. **a)** 30°-tilted SEM images of SiO_2 vertical pillar arrays of diameter, *D*, 350 nm, 450 nm, and 550 nm and a 2:1 aspect ratio (height : diameter). Scale bar, 2 μm. **b)** SEM images showing a top view of SiO_2 vertical pillar arrays of diameter, *D*, 550 nm and 2:1 aspect ratio (height : diameter) with a pillar pitch of 2 μm and 4 μm. Scale bar, 2 μm.

Table S1. Characteristics of the different SiO2 vertical pillar engineered by soft-NIL used in this study

	Pillar 350 nm	Pillar 450 nm	Pillar 550 nm 2 μm pitch	Pillar 550 nm 4 μm pitch
**Diameter (nm)**	350	450	550	550
**Height (nm)**	200	600	1100	1000
**Pitch (nm)**	1000	1000	2000	4000

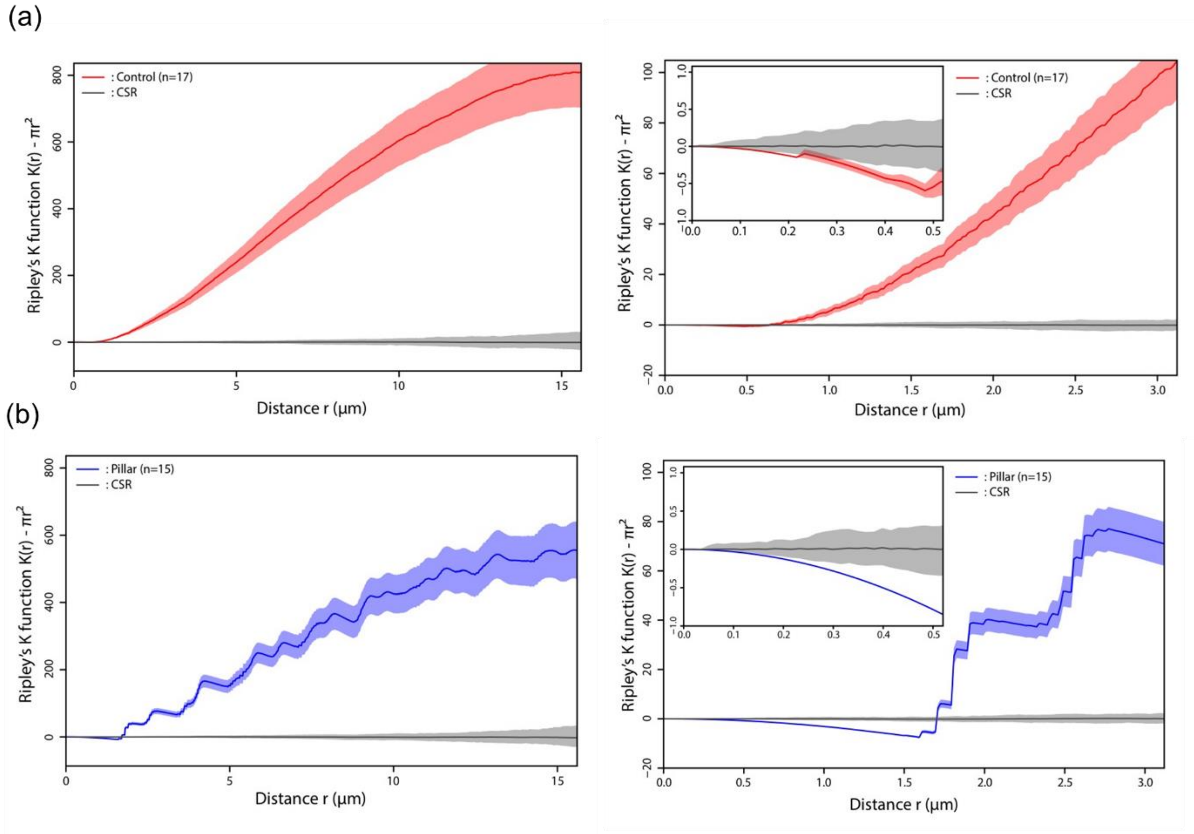

Figure S2. Computation of the Ripley's K function on flat and 550 nm diameter vertical pillars. a) Average Ripley's K function ± SEM at different length scales (*r*) of hDCs seeded on flat SiO_2 surfaces (red curve) at $r < 15$ µm, $r < 3$ µm, and $r < 0.5$ µm (inset). Black curve represents the averaged Ripley's K function of CSR simulated pattern. Gray shade represents envelope containing 95% of simulations. Cells (n) analyzed from > 3 biological replicates, n = 17. **b)** Average Ripley's K function ± SEM at different length scales (*r*) of hDCs seeded on 550 nm diameter pillar array surfaces (blue curve) at $r < 15$ µm, $r < 3$ µm, and $r < 0.5$ µm (inset). Black curve represents the averaged Ripley's K function of CSR simulated pattern. Gray shade represents envelope containing 95% of simulations. Cells (n) analyzed from > 3 independent experiments, n = 15.

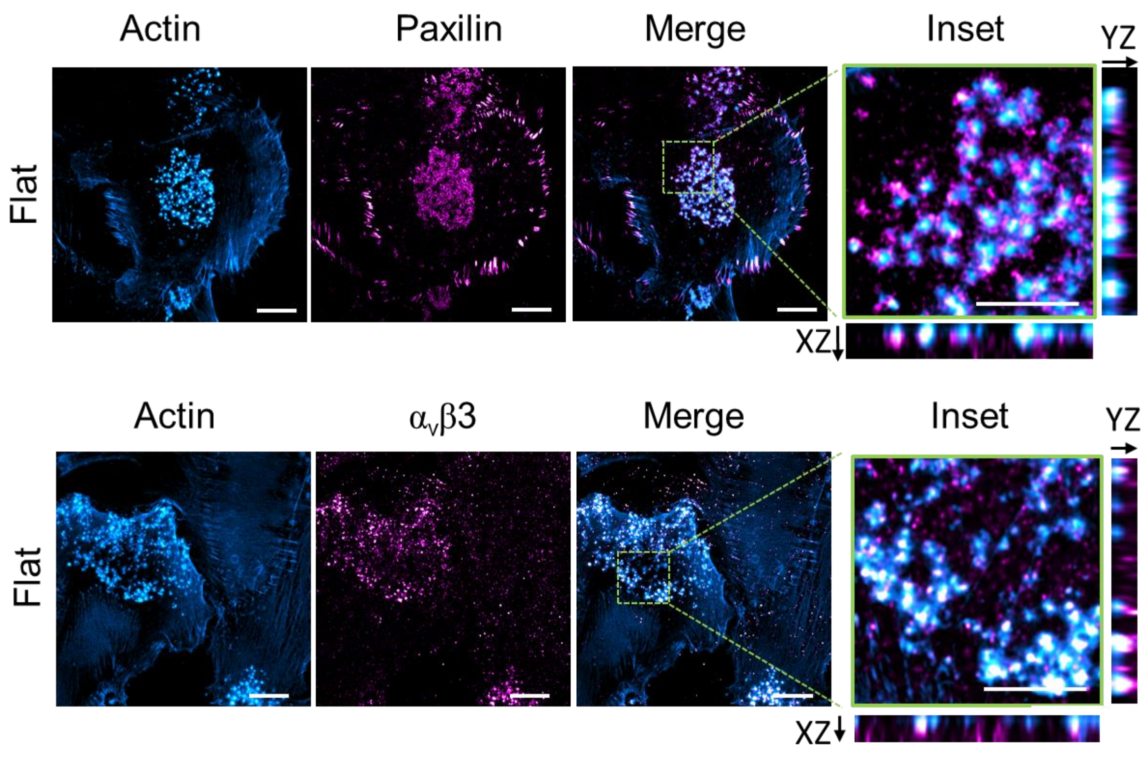

Figure S3. Immunolabeling of adhesion molecules in hDCs cultured on flat surfaces. Z-projected Airyscan images (XY plane) of hDCs cells cultured on flat surfaces showing the organization of F-actin (blue) and paxillin or αvβ3 (magenta). Orthogonal view displaying an example of XZ and YZ planes. Scale bar, 10 µm and 5 µm (inset).

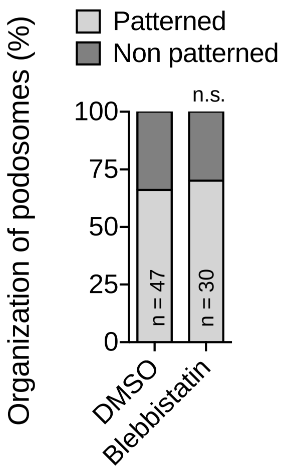

Figure S4. Effect of myosin-II on the spatial organization of podosomes. % of hDCs seeded on vertical pillar topographies showing patterned or non-patterned podosomes on control conditions (DMSO) or after 30 min treatment with blebbistatin (Myosin-II inhibitor). Chi-square test: n.s. P > 0.05. Cells analyzed (n) from ≥ 2 biological replicates are shown in the graph.

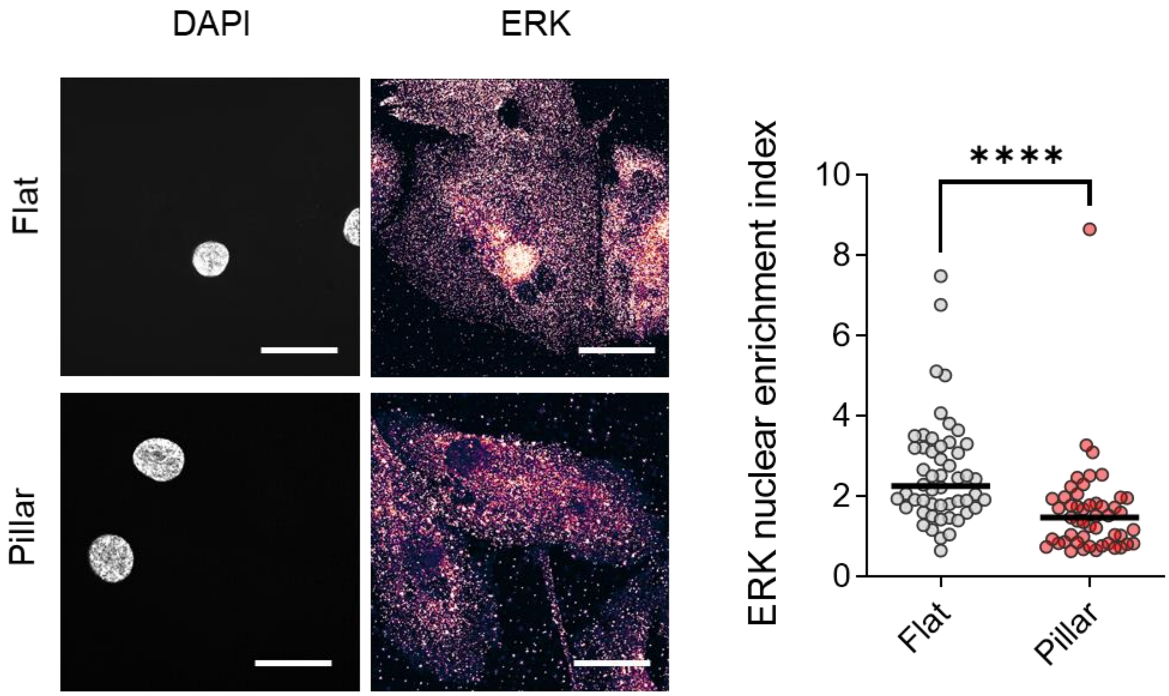

Figure S5. Nuclear enrichment of ERK as a function of the surface topography. Confocal images of endogenous ERK (fire LUT) and the nucleus (DAPI, gray) of hDCs cultured on flat or vertical pillar topographies. Scale bar, 20 µm. **f)** Quantification of the ERK nuclear enrichment of hDCs cultured on flat (gray) or vertical pillar topographies (red). Solid line represents the mean. Cells analyzed (n) from 2 biological replicates n = 52 and n = 49, respectively. Mann-Whitney test: **** $P < 0.0001$.
